# Effects of rehydration and nutritional recovery strategies after rapid weight loss on body mass recovery, hydration status, and performance in combat sports athletes: a systematic review and meta-analysis

**DOI:** 10.3389/fnut.2026.1943678

**Published:** 2026-09-18

**Authors:** Jianbo Sun, Huixin Song, Jingyuan Sun

**Affiliations:** 1 School of Health Sciences, Universiti Sains Malaysia, Kota Bharu, Kelantan, Malaysia; 2 International Korfball Development Research Center, Zhengzhou University, Zhengzhou, Henan, China

**Keywords:** carbohydrate refeeding, fluid balance, rapid weight gain, recovery monitoring, urine specific gravity, weight cutting, weight-category athletes

## Abstract

**Background:**

Rapid Weight Loss (RWL) is common in weight-category combat sports, but the extent to which post-weigh-in feeding and rehydration restore body mass, hydration, and exercise performance remains uncertain.

**Objective:**

To quantify post-weigh-in body-mass regain and hydration status and to evaluate nutritional and rehydration strategies and subsequent exercise performance in combat-sport athletes.

**Methods:**

This review followed PRISMA 2020 and was registered in PROSPERO (CRD420261438676). PubMed, SPORTDiscus, Embase, and Web of Science were searched from inception to July 2026. English-language human studies involving RWL or weight making followed by recovery were eligible. Comparable continuous outcomes were pooled using random-effects models with modified Hartung-Knapp confidence intervals; heterogeneous strategy and performance outcomes were synthesized using evidence maps and effect-direction methods.

**Results:**

Forty studies were included in the main post-weigh-in recovery review. Pooled post-weigh-in body-mass regain was 6.23% of body mass (95% CI 3.74–8.71; *k* = 11; *n* = 1504; *I*^2^ = 99.2%) and 3.71 kg (95% CI 2.45–4.96; *k* = 12; *n* = 1451; *I*^2^ = 98.9%). In five matched cohorts with paired pre-weigh-in and follow-up measurements, RWL was 8.33% of body mass (95% CI 6.17–10.49; *n* = 179), followed by a median regain of 106.5% of the preceding loss (range 63.2–154.1%). At official weigh-in, pooled mean urine specific gravity (USG) was 1.029 (95% CI 1.025–1.033; *k* = 3; *n* = 58; *I*^2^ = 92.7%). Urine osmolality was reported in seven studies and plasma osmolality in four, but these data were not sufficiently comparable for pooling. Twenty-three performance records from 12 studies were too heterogeneous for meta-analysis. No specific nutritional or rehydration approach had sufficiently homogeneous comparative evidence for a strategy-effect meta-analysis.

**Conclusion:**

Combat-sport athletes commonly regain substantial body mass after weigh-in, but body-mass regain should not be interpreted as complete rehydration or restored performance readiness. Current strategy-specific evidence is insufficient to identify a superior recovery protocol; standardized, adequately powered trials using multidimensional recovery outcomes are needed.

**Systematic review registration:**

identifier: CRD420261438676.

## 1 Introduction

Combat sports are organized around weight categories, creating strong incentives for athletes to manipulate body mass before the official weigh-in (1). Rapid weight loss (RWL) is therefore common in boxing, wrestling, judo, mixed martial arts, and related combat sports, particularly when athletes attempt to qualify for a lower weight class (2, 3). Although this practice is intended to provide a competitive size advantage after weigh-in, it can impose substantial acute physiological stress, including hypohydration, disturbed fluid and electrolyte balance, and possible impairment of high-intensity exercise capacity (1, 4, 5).

The interval between weigh-in and competition represents a critical recovery window in which athletes attempt to restore body mass, fluid balance, glycogen availability, and performance readiness (6, 7). Strategies investigated during this period include *ad libitum* eating and drinking, high-carbohydrate refeeding, carbohydrate-containing beverages, creatine plus glucose, glycerol-based rehydration, caffeine, sodium-containing supplements, and structured weight-management protocols (8–15). However, these recovery endpoints are not interchangeable. Athletes may regain substantial body mass while remaining hypohydrated or incompletely recovered in neuromuscular, repeated-effort, or glycogen-related outcomes (8, 16).

Previous reviews have described the prevalence, magnitude, and methods of RWL and rapid weight gain in combat sports, together with their potential health and performance consequences (2–5). However, the post-weigh-in recovery phase has received less integrated synthesis. Individual studies differ substantially in sport, weigh-in regulations, recovery duration, recovery strategy, hydration assessment, and performance testing. Consequently, it remains uncertain how much body mass athletes typically regain, whether hydration status normalizes before competition, and whether specific nutritional or rehydration strategies improve subsequent performance.

A further concern is that body-mass regain is frequently interpreted as evidence of physiological recovery. This assumption may be misleading because body mass, hydration status, muscle glycogen, and performance capacity can recover at different rates (8, 17, 18). Separating these endpoints is important for both research interpretation and applied practice: a strategy that increases body mass may not necessarily restore USG, plasma or urine osmolality, blood volume, perceived readiness, or sport-relevant performance. In field practice, USG <1.020 is commonly used as an operational criterion for euhydration, whereas values ≥1.020 indicate hypohydration and values approaching or exceeding 1.030 reflect marked urine concentration; these thresholds should be interpreted alongside sampling time and recent fluid or solute intake (1, 19).

Therefore, this systematic review and meta-analysis aimed to evaluate post-weigh-in rehydration and nutritional recovery following RWL in combat-sport athletes. The primary outcomes were body-mass regain after weigh-in and hydration status around weigh-in and recovery. Secondary outcomes included exercise performance, prevalence of hypohydration, mechanistic and safety-related markers, and the evidence supporting specific recovery strategies. Where quantitative synthesis was feasible, random-effects meta-analysis was performed; where outcomes were too heterogeneous, structured narrative synthesis and evidence-direction mapping were used.

## 2 Methods

### 2.1 Protocol and reporting

This systematic review and meta-analysis was conducted and reported in accordance with the Preferred Reporting Items for Systematic Reviews and Meta-Analyses (PRISMA 2020) statement (20). The review was registered in PROSPERO on 7 July 2026 (CRD420261438676), after the review commenced on 1 July 2026 but before formal screening, data extraction, risk-of-bias assessment, and data synthesis.

Several deviations from the registered record occurred. Eligibility was broadened from randomized studies to include non-randomized interventions and observational studies because randomized comparative evidence was sparse and the review also aimed to quantify real-world post-weigh-in body-mass and hydration outcomes. The final search was limited to English-language reports and to PubMed, Embase, Web of Science, and SPORTDiscus; SPORTDiscus was added for sport-specific coverage, whereas the planned searches of ASSIA, CENTRAL, CINAHL, Google Scholar, standalone MEDLINE, study registers, and dissertation databases were not undertaken. Reference-list checking was retained, but forward citation searching, author contact, and acquisition of individual-participant datasets were not undertaken.

RoB 2 was retained for randomized and crossover studies, whereas ROBINS-I was used for non-randomized studies after the eligibility expansion. Formal outcome-level GRADE ratings and formal tests of funnel-plot asymmetry were not completed because the principal syntheses were highly heterogeneous single-arm pooled means or non-poolable outcomes. Compatible study designs were combined only for descriptive single-arm means, not to estimate causal intervention effects. Descriptive single-arm pooling, evidence maps, effect-direction plots, exploratory restriction and sensitivity analyses, and the salt-bath comparison were added after inspection of the available data and were treated as descriptive, supplementary, or exploratory. Planned subgroup or risk-of-bias-restricted analyses were not conducted when data were insufficient or no comparable low-risk subset was available.

### 2.2 Eligibility criteria

English-language original human studies were eligible if they investigated athletes from weight-category combat sports, including boxing, wrestling, judo, mixed martial arts, kickboxing, taekwondo, karate, Muay Thai, and related sports. Participants could be amateur, collegiate, elite, or professional athletes of any sex, provided that the study involved RWL, weight making, or an official or simulated weigh-in followed by a recovery period.

Eligible exposures or interventions included post-weigh-in rehydration, nutritional recovery, *ad libitum* eating or drinking, carbohydrate refeeding, carbohydrate-containing beverages, creatine plus glucose, glycerol-based rehydration, caffeine, sodium-containing supplements, water loading, and structured weight-management protocols. Observational competition studies were also eligible when they reported body-mass regain, hydration status, performance outcomes, or relevant mechanistic or safety-related outcomes after weigh-in. Studies focused only on RWL methods were excluded from the main recovery-strategy synthesis but were retained for prespecified supplementary or exploratory analyses when they provided relevant RWL-magnitude or weight-loss-method data.

The primary outcomes were post-weigh-in body-mass regain, expressed as percentage of body mass or absolute kilograms, and hydration status around weigh-in or recovery, primarily assessed using USG. Secondary outcomes included RWL magnitude, hypohydration prevalence, exercise performance, recovery or nutritional strategies, and mechanistic or safety-related markers. Reviews, commentaries, conference abstracts without extractable data, non-human studies, studies in non-combat sports, and studies without relevant RWL, recovery, hydration, body-mass, or performance outcomes were excluded. When multiple reports appeared to describe overlapping participants, the most complete or most relevant dataset was retained for each analysis.

### 2.3 Search strategy and study selection

A systematic literature search was conducted in PubMed, Embase, Web of Science, and SPORTDiscus EBSCOhost from database inception to July 2026. The search strategy combined terms related to combat sports, RWL, weigh-in, rehydration, nutritional recovery, hydration status, and performance. The hydration block explicitly included USG, urine osmolality, and plasma osmolality because eligible studies used different biomarkers of hydration. The full search strategy for each database is provided in Supplementary Table S1. Reference lists of relevant reviews and eligible full-text articles were also checked to identify additional studies.

All retrieved records were exported to a screening spreadsheet, and duplicates were removed before screening. Two reviewers independently screened titles and abstracts against the predefined eligibility criteria and then assessed potentially eligible full texts. Disagreements were resolved through discussion; when consensus could not be reached, a third reviewer made the final decision.

### 2.4 Data extraction

Data were extracted using a prespecified standardized form in accordance with established systematic-review guidance (21). Two reviewers independently extracted and cross-checked the data, and disagreements or uncertainties were resolved through discussion or consultation with a third reviewer.

For each study, the extracted information included first author, publication year, sport, country or competition setting where available, study design, participant characteristics, sample size, sex, competitive level, weigh-in context, recovery duration, rapid weight-loss method, recovery strategy, comparator, and relevant outcomes. Recovery strategies were classified as *ad libitum* eating or drinking, carbohydrate refeeding, carbohydrate-containing beverages, creatine plus glucose, glycerol-based rehydration, caffeine, sodium-containing supplements, water loading, structured weight-management protocols, or other relevant strategies.

For quantitative synthesis, means, standard deviations, sample sizes, confidence intervals, event counts, percentages, and relevant test statistics were extracted where available. Body-mass outcomes were extracted as both kilograms and percentage of body mass when reported, but these metrics were analyzed separately. Outcomes measured at different time points, including official weigh-in, post-recovery, pre-competition, and post-competition, were extracted separately and were not collapsed unless they represented the same clinical or practical phase. For overlapping datasets, subgroup and total-sample data were not entered simultaneously into the same meta-analysis.

### 2.5 Risk of bias assessment

Risk of bias was assessed according to study design. Randomized and crossover intervention studies were assessed using the Cochrane Risk of Bias 2 framework (22), with attention to the randomization process, deviations from intended interventions, missing outcome data, outcome measurement, and selective reporting. For crossover studies, additional consideration was given to washout periods, potential carryover effects, period effects, and whether within-participant comparisons were appropriately handled.

Non-randomized intervention studies, observational field studies, and retrospective competition datasets were assessed using domains adapted from ROBINS-I (23). The main domains considered were confounding, participant selection, exposure or intervention classification, deviations from intended exposure, missing data, outcome measurement, and selective reporting. For real-world weigh-in or competition studies, particular attention was given to confounding by sport, sex, weight category, competitive level, recovery window, weigh-in rules, and self-selected weight-making practices.

Risk-of-bias assessments were independently completed by two reviewers. Disagreements were resolved through discussion, and unresolved disagreements were adjudicated by a third reviewer. Formal overall judgements were summarized for the 40 studies in the main recovery synthesis; the 16 supplementary or contextual studies did not contribute to the primary pooled estimates or strategy-effect conclusions and were not included in the overall risk-of-bias summary. Main-synthesis studies were classified as low risk, some concerns or moderate risk, or high risk of bias according to the overall judgement across domains.

### 2.6 Statistical analysis

Meta-analyses were conducted when at least two studies reported sufficiently comparable quantitative data for the same outcome and assessment period. Post-weigh-in body-mass regain, rapid weight-loss magnitude, and urine specific gravity (USG) were analyzed as single-arm continuous outcomes and expressed as pooled means with 95% confidence intervals (CIs). Relative body-mass change (%BM) and absolute body-mass change (kg) were analyzed separately. Hydration measurements obtained at substantively different phases, including official weigh-in, post-recovery, and pre-competition assessments, were not combined.

Study-level standard errors were calculated from reported standard deviations and sample sizes. Because clinical and methodological heterogeneity was anticipated, raw means were pooled using DerSimonian–Laird random-effects models (24), with modified Hartung–Knapp CIs to provide more conservative inference when few studies or studies of unequal precision were available (25). Statistical heterogeneity was assessed using the between-study variance (τ^2^), Cochran's Q, and the *I*^2^ statistic (26). Prediction intervals were calculated for syntheses containing at least three studies to describe the expected range of values in a comparable future study, although these intervals were interpreted cautiously when only a small number of studies was available (27).

Exploratory subgroup analyses were conducted according to sport, recovery duration, and study setting when sufficient data were available. To examine the robustness of the primary results to statistical assumptions, sensitivity analyses replaced the DerSimonian–Laird estimator with restricted maximum likelihood and Paule–Mandel estimators and compared conventional and modified Hartung–Knapp CIs, as well as fixed-effect and random-effects estimates. Additional sensitivity analyses excluded derived or combined-subgroup estimates, retrospective cohorts, the structured weight-management protocol, samples with fewer than 10 participants, and recovery windows of at least 30 h. Prospective competition-only, sport-restricted, and leave-one-out analyses were also performed where feasible. For the USG synthesis, additional analyses examined the effects of excluding the smallest study, including an experimental post-RWL measurement, and sequentially omitting each study. Risk-of-bias-restricted analyses were planned when a sufficiently large and methodologically comparable low-risk subset was available.

Low-hydration prevalence was not pooled when studies differed materially in biomarkers, thresholds, denominators, or assessment times. Strategy-specific effects were pooled only when interventions, comparators, outcomes, and assessment periods were sufficiently comparable. Salt-water vs. fresh-water hot-water immersion was treated as an exploratory analysis of rapid weight-loss methods rather than as an analysis of post-weigh-in nutritional recovery.

Funnel plots were generated for body-mass-regain syntheses containing at least 10 estimates and were interpreted as descriptive indicators of possible small-study effects rather than as direct evidence of publication bias (28). Formal tests of funnel-plot asymmetry were not performed because the analyses involved single-arm pooled means and substantial clinical and statistical heterogeneity.

Exercise-performance outcomes were not quantitatively pooled because the included studies used heterogeneous tests, intervention contrasts, effect measures, and recovery periods. These outcomes were therefore examined using effect-direction plots. A study-level sensitivity analysis was also conducted so that studies reporting multiple performance tests or assessment times did not contribute disproportionate weight to the synthesis.

All analyses were conducted using Python 3.12.13, pandas 3.0.1, NumPy 2.3.5, matplotlib 3.11.0, and purpose-developed scripts.

### 2.7 Narrative and evidence-direction synthesis

When quantitative pooling was inappropriate, findings were synthesized using structured narrative methods informed by the Synthesis Without Meta-analysis (SWiM) reporting guideline (29). Studies were grouped according to prespecified outcome domains: body-mass outcomes, hydration outcomes, recovery or nutritional strategies, exercise performance, mechanistic markers, and safety-related outcomes. Effect-direction plots were constructed in accordance with published guidance for the transparent synthesis and visualization of heterogeneous outcomes (30).

Low-hydration prevalence was summarized according to the biomarker used, applied threshold, denominator, and measurement time point. Performance outcomes were grouped into strength or neuromuscular performance, repeated high-intensity effort, and endurance or fatigue-related outcomes. Records were additionally classified according to whether they represented a nutritional or rehydration intervention, an RWL or dehydration exposure, or an assessment conducted during the post-weigh-in recovery period.

For each performance outcome, effect direction was determined primarily from the direction of the reported point estimate, between-condition contrast, or within-participant change. Outcomes were classified as improved or recovered, unchanged or showing no clear directional difference, impaired, or mixed or unclear. Statistical significance and the original authors' interpretation were recorded as supporting information but were not used alone to determine effect direction (30). When a study reported multiple tests or assessment times within the same performance domain, these records were displayed separately in the outcome-level plot but consolidated in the study-level sensitivity analysis.

Recovery and nutritional strategies were summarized using an evidence map showing the strategy evaluated, outcome domain, study design, and direction of findings. Strategies were not classified as effective or ineffective unless comparable quantitative evidence was available from more than one study. Mechanistic and safety-related outcomes, including urine or plasma osmolality, plasma volume, total body water, renal or urinary markers, hormonal responses, oxidative stress, metabolic markers, perceptual responses, and competition outcomes, were narratively synthesized to determine whether body-mass regain was accompanied by recovery of hydration, physiological status, or performance readiness.

## 3 Results

### 3.1 Study selection and study characteristics

The database search identified 980 records from PubMed (*n* = 206), SPORTDiscus (*n* = 44), Embase (*n* = 253), and Web of Science (*n* = 477). After removal of 216 duplicates, 764 records underwent title and abstract screening; 686 were excluded and 78 reports were sought for retrieval. Seven reports could not be retrieved, leaving 71 full-text reports for eligibility assessment. Of these, 40 met the eligibility criteria for the main post-weigh-in recovery review. The remaining 31 reports did not enter the main review because they were outside the main recovery-review scope (*n* = 22), focused on measurement or validation (*n* = 7), or were conference abstracts or reports with insufficient information (*n* = 2). All 40 main-review studies arose from database records; reference-list checking identified no additional eligible studies (Figure 1). As a supplementary note, 15 reports outside the main-review set were used for context-specific hydration or prevalence, performance effect-direction, or mechanistic and safety syntheses, and one case study was used only for narrative context. These 16 reports were not counted among the 40 studies in the main review; their analytical roles are specified separately in Supplementary Table S2.

**Figure 1:**
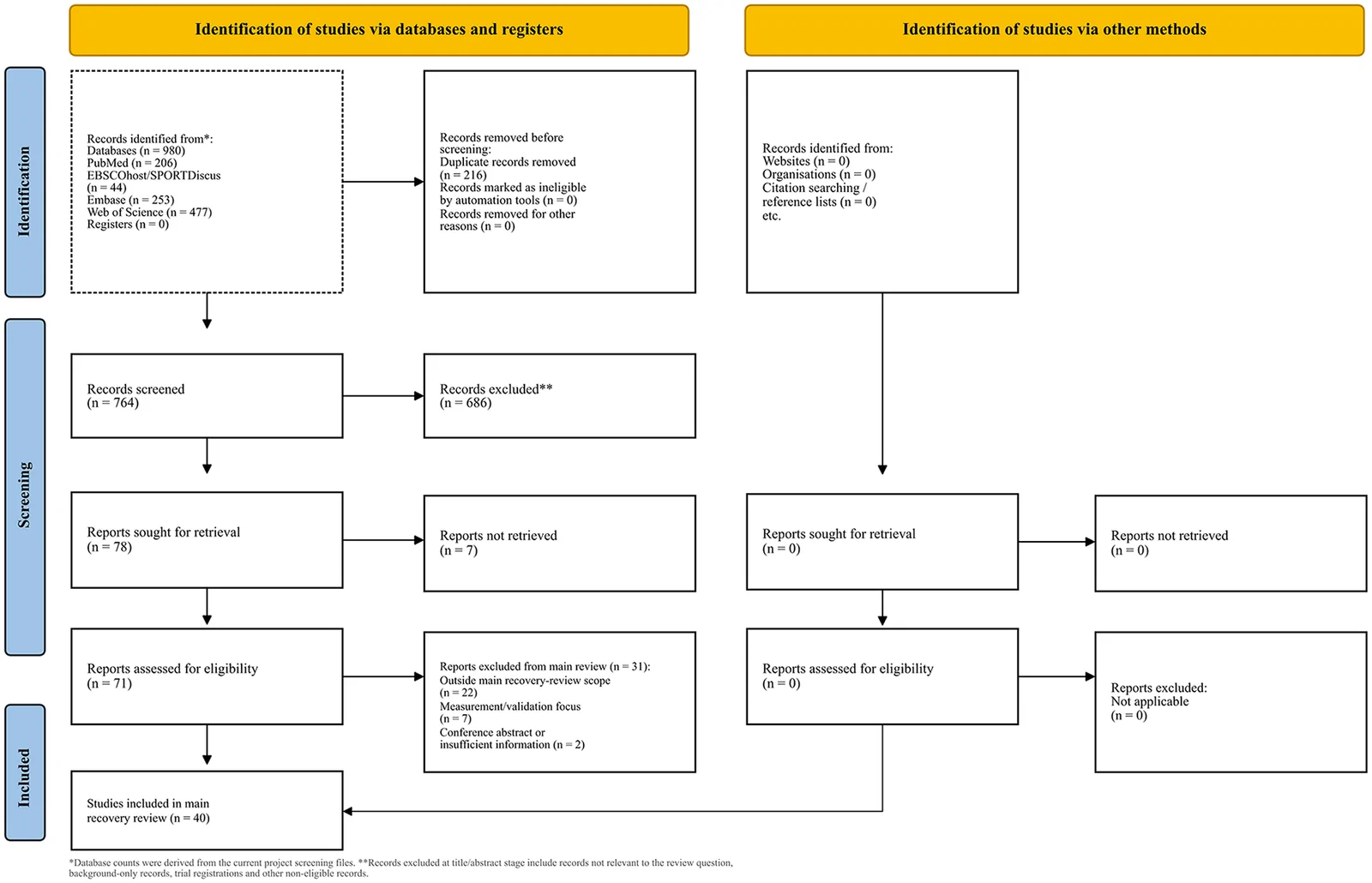
PRISMA 2020 flow diagram for the main post-weigh-in recovery review. Forty studies met the main-review eligibility criteria and were included.

The 40 main-review studies investigated wrestling, judo, boxing, mixed martial arts, taekwondo, kickboxing or Muay Thai, and mixed combat-sport samples. They comprised real-world weigh-in or competition observations, repeated-measures field studies, randomized or crossover interventions, laboratory RWL protocols, and structured weight-management interventions. Recovery windows ranged from several hours to overnight, 16–24 h, and competition-day assessments. Sex reporting was incomplete in several reports; Supplementary Table S2 records sex exactly as reported and denotes unreported information as NR. Characteristics and analytical roles are presented in Supplementary Table S2.

### 3.2 Risk of bias in included studies

Among the 40 studies included in the main recovery review, none was judged to be at low risk of bias across all domains (8–18, 31–59). Fourteen studies were rated as having some concerns, 21 were judged to have moderate-to-serious risk of bias, three were judged to have serious risk of bias, and two single-arm or small-sample studies were judged to have serious limitations and were not considered suitable for quantitative meta-analysis.

The main sources of bias reflected the nature of the available evidence. Many studies were real-world weigh-in or competition observations, in which RWL, recovery duration and post-weigh-in intake were self-selected rather than randomized. These studies were therefore vulnerable to confounding by sport, sex, weight category, competitive level, weigh-in rules, opponent characteristics and recovery window. In intervention and crossover studies, common concerns included small sample sizes, limited blinding, incomplete reporting of randomization or washout procedures, and heterogeneity in recovery protocols and performance tests.

Risk-of-bias judgements were used to guide interpretation rather than to exclude studies automatically. Studies with serious methodological limitations were retained when they provided relevant field evidence, but their findings were interpreted cautiously and were generally handled in narrative or supplementary analyses rather than as definitive intervention effects. Detailed domain-level judgements are presented in Supplementary Figure S1.

### 3.3 Body-mass regain after weigh-in

Body-mass regain was reported in 12 of the 40 main-review studies. One additional renal and urinary safety report (60) also provided extractable RWL and body-mass-regain data, yielding 13 contributors to the quantitative body-mass dataset; 11 contributed relative-regain data, 12 contributed absolute-regain data, and 10 contributed to both analyses. The pooled post-weigh-in increase was 6.23% of body mass (95% CI 3.74–8.71; *k* = 11; *n* = 1,504; Figure 2A) and 3.71 kg (95% CI 2.45–4.96; *k* = 12; *n* = 1,451; Figure 2B). Heterogeneity was considerable for both relative (*I*^2^ = 99.2%) and absolute regain (*I*^2^ = 98.9%). Exploratory funnel plots showed broad dispersion (Supplementary Figure S2).

**Figure 2:**
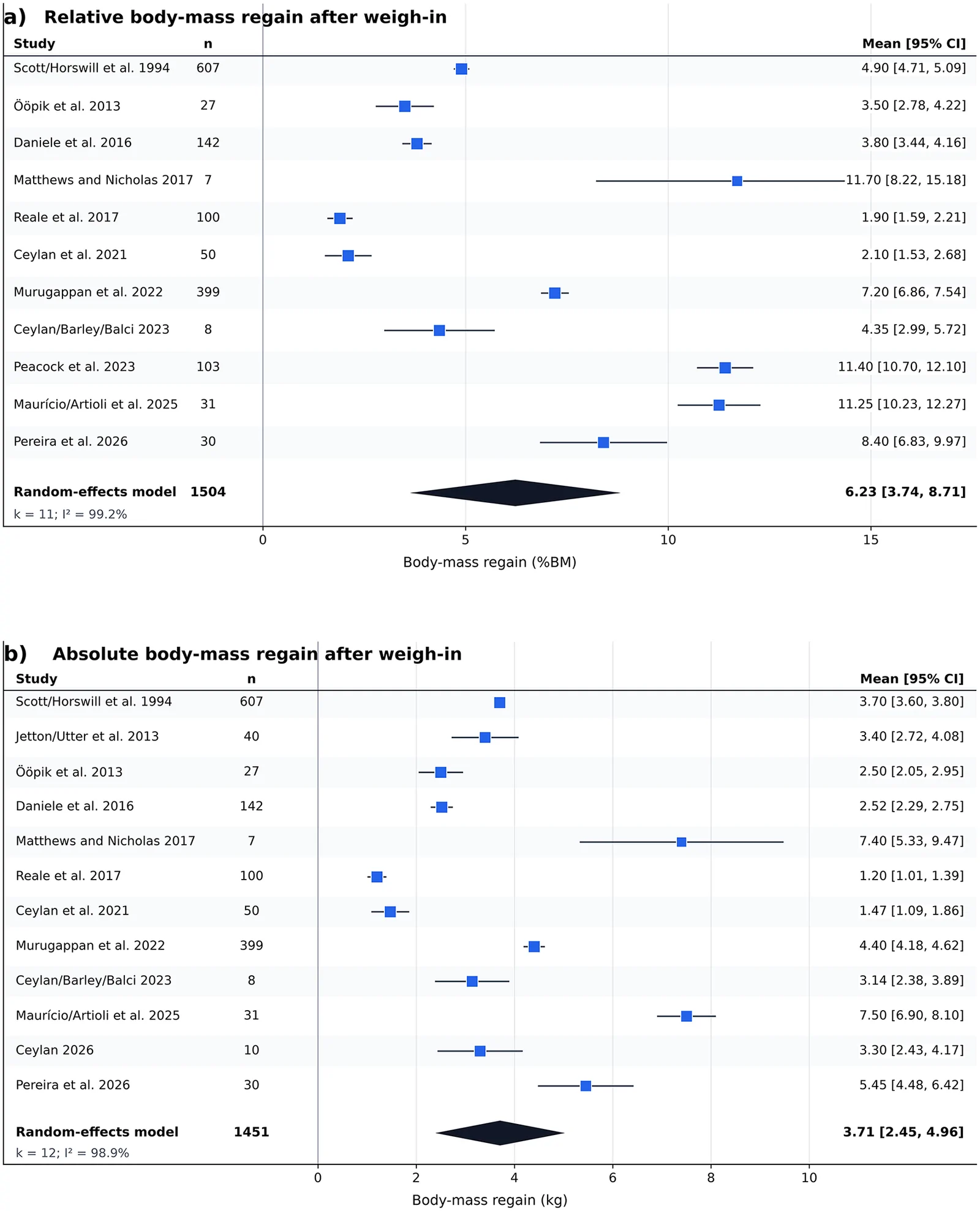
Body-mass regain after weigh-in. **(A)** Relative body-mass regain expressed as percentage of body mass (%BM). **(B)** Absolute body-mass regain expressed in kilograms (kg).

Across leave-one-out analyses, relative regain ranged from 5.63 to 6.67%, while absolute regain ranged from 3.33 to 3.92 kg. Alternative between-study variance estimators produced similar pooled means; the fixed-effect estimate for relative regain was 4.75%, compared with 6.23% in the primary random-effects model. Excluding recovery windows of at least 30 h reduced pooled regain to 5.25% of body mass and 3.08 kg, and restriction to boxing and grappling studies produced estimates of 3.97% and 2.70 kg, respectively. Prediction intervals were −0.07 to 12.52% for relative regain and 0.30 to 7.11 kg for absolute regain (Supplementary Figure S3).

Where pre-weigh-in RWL was extractable, the pooled reduction was 8.33% of body mass (95% CI 6.17–10.49; *k* = 5; *n* = 179; *I*^2^ = 95.1%). Leave-one-out estimates ranged from 7.47 to 8.71%. Exclusion of the structured weight-management protocol reduced the estimate to 7.47% (95% CI 6.40–8.53; *I*^2^ = 46.9%). The prediction interval was 2.49–14.16%.

Five studies reported pre-weigh-in loss and subsequent regain in the same cohort (*n* = 179). Athletes regained 63.2–154.1% of the preceding loss, with a median recovery fraction of 106.5%. At follow-up, body mass was above the pre-RWL value in three studies and remained below it in two. These data were summarized descriptively because within-study covariance was unavailable.

The five matched cohorts used non-equivalent observation windows. Pre-weigh-in loss was measured over the final 72 h, 7 days, or from a longer precompetition baseline, with athletes in one study initiating weight cutting 8–29 days before weigh-in. Post-weigh-in follow-up was generally approximately 24–32 h; one judo cohort was measured 24 h after competition rather than immediately before it (Supplementary Table S3).

### 3.4 Hydration status around weigh-in and recovery

Urine specific gravity was reported in 16 of the 40 main-review studies. Two additional reports reserved for context-specific synthesis also reported USG and are listed separately in Supplementary Table S2. Across studies, measurements were obtained at official weigh-in, experimental post-RWL, post-rehydration, precompetition, competition-day, and weight-management time points. Only three real-world competition studies provided sufficiently comparable official-weigh-in means for the primary quantitative synthesis; their pooled mean was 1.029 (95% CI 1.025–1.033; *k* = 3; *n* = 58; *I*^2^ = 92.7%; Figure 3A).

**Figure 3:**
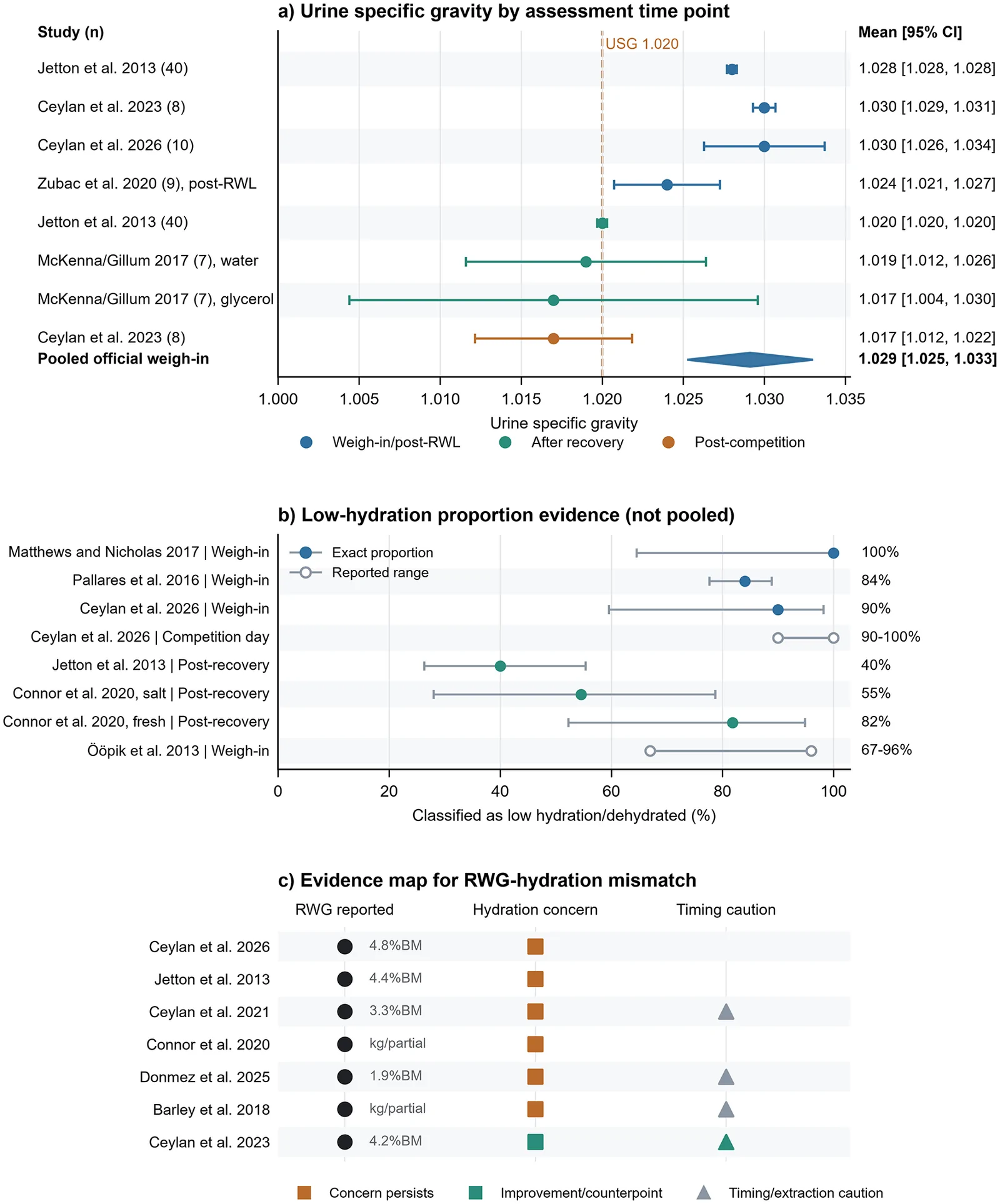
Hydration status around weigh-in and recovery. **(A)** Study-level USG estimates at official weigh-in (*k* = 3), in the official-weigh-in plus experimental post-RWL sensitivity set (*k* = 4), and at two later single-study assessments (*k* = 1 at each time point). **(B)** Study-level prevalence of hypohydration using reported thresholds. **(C)** Alignment of body-mass and hydration findings by assessment phase.

When official-weigh-in and experimental post-RWL measurements were considered together, the pooled mean USG was 1.028 (95% CI 1.024–1.032; *k* = 4; *n* = 67; *I*^2^ = 91.2%).

After recovery, one study reported a mean USG of 1.020 before competition (*n* = 40), and one study reported a mean USG of 1.017 at a 24-h post-weigh-in or post-competition assessment (*n* = 8). Hypohydration-prevalence studies used different thresholds and sampling windows and were therefore summarized descriptively (Figure 3B).

Urine osmolality was reported in seven studies and plasma osmolality in four. Measurements spanned official weigh-in, experimental post-RWL, post-rehydration, precompetition, and competition-related time points and used different contrasts and reporting formats; these data were not quantitatively pooled. Figure 3C aligns the available body-mass and hydration measurements by assessment phase.

### 3.5 Recovery and nutritional strategy evidence

Ten recovery or weight-management strategy categories were identified; categories were not mutually exclusive. *Ad libitum* eating and drinking was represented in seven studies, high-carbohydrate refeeding or recovery meals in five recovery studies plus one RWL-context study, carbohydrate-containing or sports drinks in three studies, and structured weight-management protocols in two studies. Creatine plus glucose, glycerol plus water, caffeine, and sodium citrate were each evaluated in one study. Water loading and salt vs. fresh-water hot bathing were each evaluated in two studies and were retained as exploratory RWL-method evidence.

The protocols differed markedly. One carbohydrate-refeeding study provided 1.5 g/kg carbohydrate as a 25% glucose-polymer solution plus 0.5 L water. Two rehydration studies used 6% carbohydrate beverages (60 g/L) in a volume equal to 100% of body-mass loss; one formulation also contained sodium 20 mmol/L and potassium 3.2 mmol/L. An overnight high-carbohydrate protocol provided 7.1 +/−0.2 g/kg carbohydrate over 13 h, together with 4,076 +/−589 mg sodium and 0.9 g/kg protein.

The creatine crossover study compared 320 g glucose with 320 g glucose plus 30 g creatine monohydrate during 17 h of recovery, administered in four portions with fruit juice. The glycerol study compared 26 mL/kg water with 1 g/kg glycerol plus 26 mL/kg water, the caffeine study provided 6 mg/kg during the third hour of a 4-h recovery period, and the sodium-citrate study provided 600 mg/kg over a 16-h recovery period.

The water-loading protocol compared 100 with 40 mL/kg/day before both groups restricted intake to 15 mL/kg/day. In the exploratory salt-bath comparison, the pooled mean difference in body-mass loss was −0.04 kg (95% CI −0.52–0.44; *I*^2^ = 0.0%) across two small crossover studies (Figure 4).

**Figure 4:**
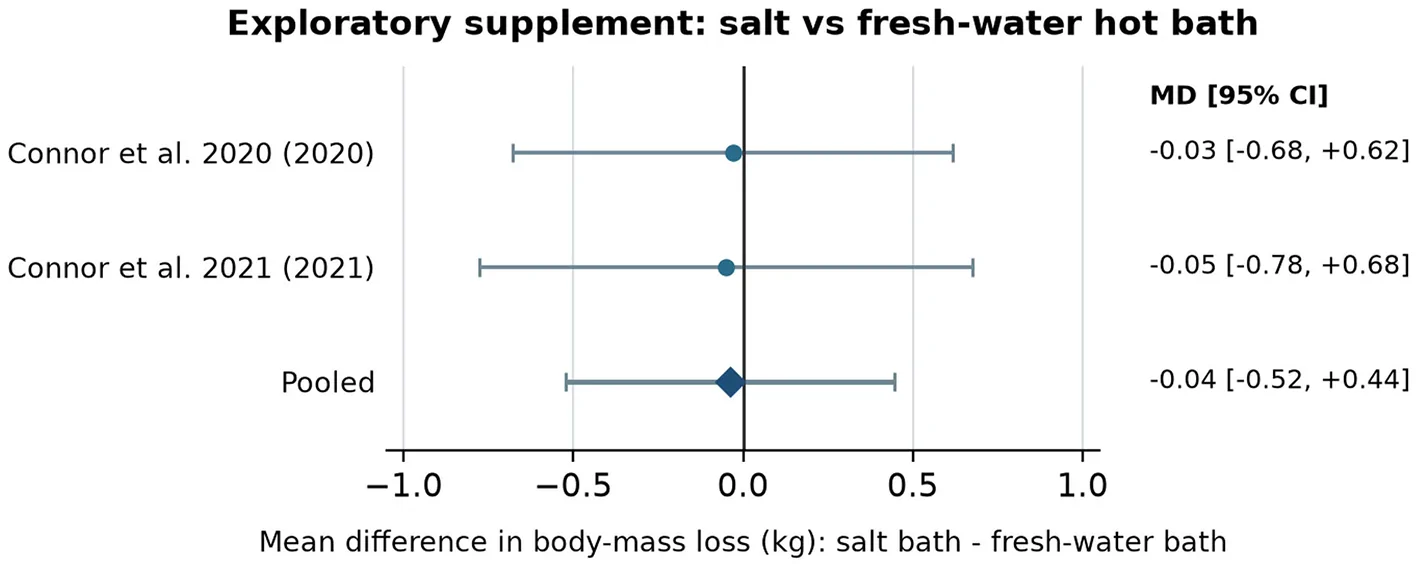
Exploratory comparison of salt-water vs. fresh-water hot bathing for acute body-mass loss.

No formal strategy-effect meta-analysis was undertaken because intervention doses, comparators, recovery windows, and outcome definitions were not sufficiently comparable. Strategy-outcome coverage and study design are displayed in the evidence map (Figure 5).

**Figure 5:**
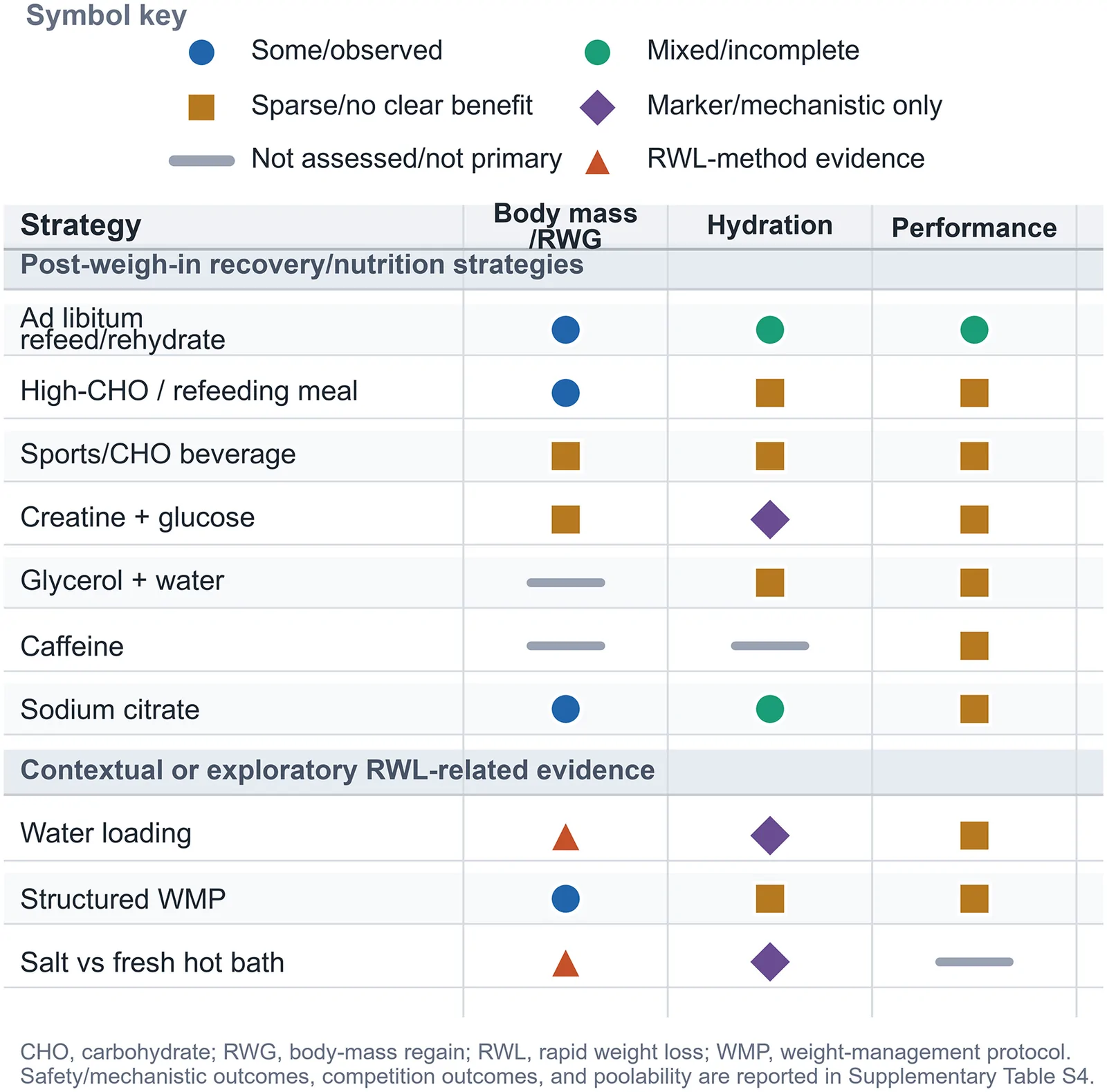
Strategy-outcome evidence map for recovery and nutrition-related approaches. Strategy categories may overlap within studies; study counts for each category are reported in the Results. Detailed study-level evidence, mechanistic and safety outcomes, competition outcomes, and poolability are reported in Supplementary Table S4.

### 3.6 Exercise performance outcomes

Exercise-performance outcomes contributed 23 effect-direction records from 12 studies across 17 indicator families. Of these, 10 records from five studies assessed strength or neuromuscular performance, 12 records from nine studies assessed high-intensity or repeated-effort performance, and one record from one study assessed endurance or fatigue time; studies could contribute to more than one domain. No performance outcome had sufficiently comparable tests, contrasts, recovery windows, and extractable effect measures for quantitative pooling (Figure 6).

**Figure 6:**
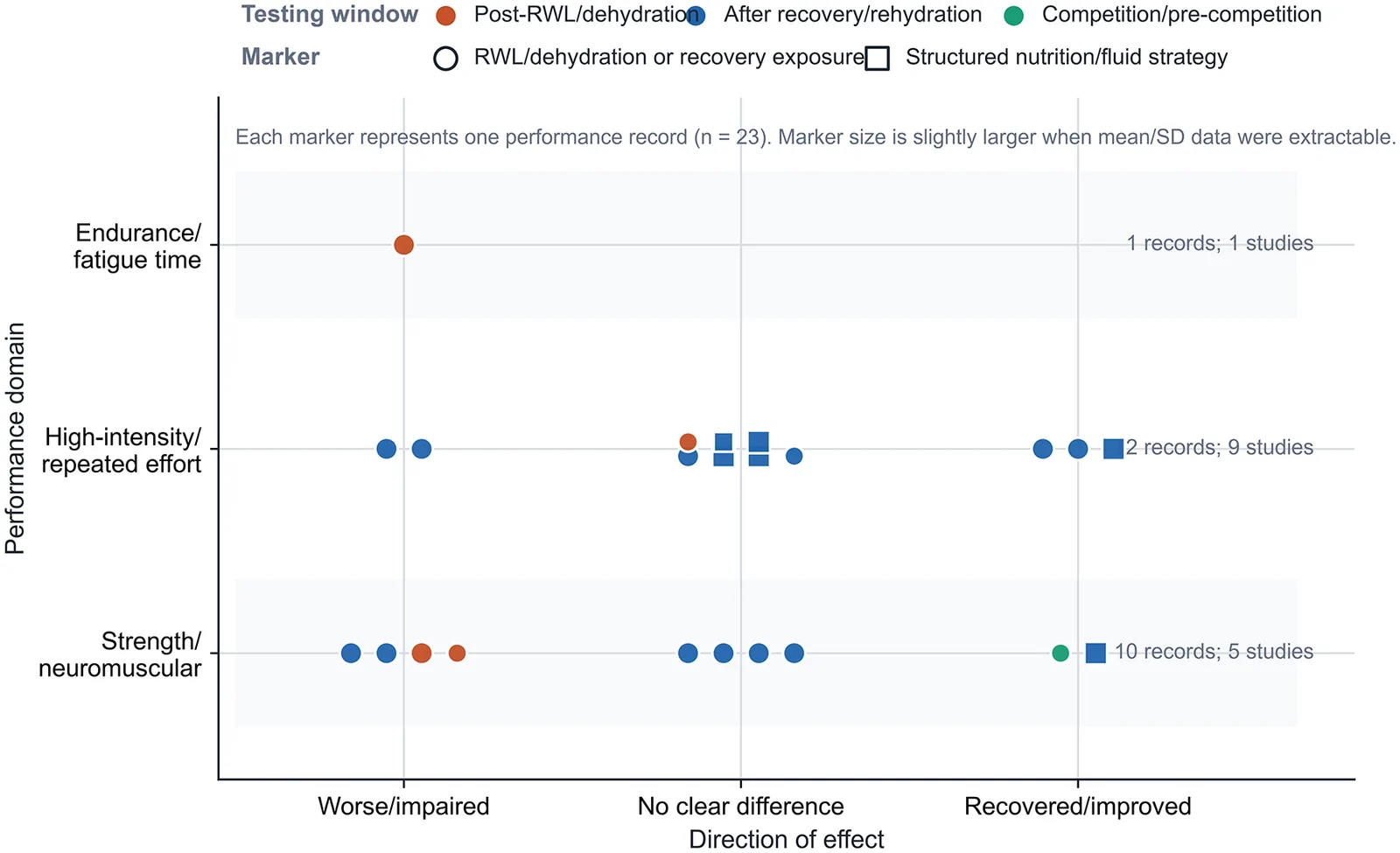
Exercise performance effect-direction harvest plot. Each marker represents one performance record from the included studies. Performance outcomes were not pooled because tests, recovery windows, intervention contrasts, and reporting formats were not sufficiently comparable.

In the study-level sensitivity synthesis, three studies were classified as showing recovery or improvement, six as mixed or showing no clear difference, and three as showing impairment. Outcome-level and study-level directions are presented in Figure 6.

### 3.7 Mechanistic and safety-related outcomes

Mechanistic and safety-related evidence comprised hydration-marker data from 15 studies, fluid or body-compartment outcomes from five studies, metabolic or glycogen outcomes from five studies, endocrine or oxidative-stress outcomes from three studies, and renal or urinary safety outcomes from one study. Categories could overlap within studies.

The five fluid or body-compartment studies reported plasma volume, total body water, extracellular water, fat-free mass, or bioelectrical-impedance-derived variables at different phases of RWL and recovery. The five metabolic or glycogen studies reported muscle glycogen, substrate oxidation, glucose availability, blood lactate, or buffering-related markers.

In individual supplement studies, sodium citrate was associated with greater body-mass and plasma-volume restoration and altered blood-buffering markers, caffeine was associated with lower perceived exertion and higher blood lactate without improved sport-specific performance, and glycerol did not produce a clear difference in acute hydration indices or anaerobic power. Three studies reported endocrine or oxidative-stress outcomes, and one 30-athlete study reported renal and urinary markers across baseline, official weigh-in, and competition-day assessments.

Adverse-event reporting was sparse. Hot-water immersion studies reported acute body-mass loss and blood or plasma-volume markers, while water-loading and structured weight-management studies reported multi-component physiological responses. These studies were retained as contextual or exploratory evidence because they did not isolate a post-weigh-in nutrient or fluid effect.

## 4 Discussion

### 4.1 Principal findings

This systematic review and meta-analysis produced three main findings. First, combat-sport athletes regained a substantial amount of body mass after weigh-in, with pooled increases of 6.23% of body mass and 3.71 kg. However, heterogeneity and prediction intervals were large, indicating that these values are descriptive averages across different sports and competition settings rather than universal recovery targets. Second, body-mass regain did not consistently correspond to restoration of hydration status. The pooled official-weigh-in USG was 1.029, while post-recovery USG evidence was limited to two single-study time points; urine and plasma osmolality evidence was also heterogeneous. Third, the evidence for individual recovery strategies was fragmented, and performance findings did not demonstrate a reproducible advantage for any specific intervention.

### 4.2 Body-mass regain and hydration recovery

The pooled magnitude of body-mass regain is consistent with previous evidence showing considerable rapid weight gain across combat sports, particularly when athletes are allowed longer recovery periods between weigh-in and competition. Matthews et al. (3) reported recovery windows ranging from 3 to 32 h and concluded that sport, competition structure and recovery duration influenced the magnitude of regain. The present analysis extends those findings by providing pooled estimates and by examining RWL and subsequent regain within matched cohorts.

Among the five matched studies, athletes regained 63.2–154.1% of the preceding body-mass loss, with a median recovery fraction of 106.5%. The observation windows were not equivalent: loss was measured over the final 72 h, 7 days, or from a longer precompetition baseline, whereas follow-up was generally approximately 24–32 h and occurred after competition in one cohort (Supplementary Table S3). Values exceeding 100% should not be interpreted as physiological over-recovery. Acute body-mass changes may reflect ingested fluid, gastrointestinal contents, glycogen-associated water, and food intake rather than complete restoration of intracellular and extracellular fluid compartments (1, 61).

Recovery duration appeared to contribute to between-study variability. Excluding recovery periods of at least 30 h reduced pooled regain to 5.25% of body mass and 3.08 kg, while restriction to boxing and grappling studies also produced lower estimates. Nevertheless, heterogeneity remained substantial, suggesting that recovery time alone did not explain the observed variation. Differences in the initial magnitude and method of RWL, weigh-in regulations, competitive level, sex, dietary intake and measurement timing probably contributed. This interpretation is consistent with previous reviews emphasizing that weight-making practices and recovery opportunities are highly sport-specific (1, 2, 6).

The hydration results provide an important counterpoint to the body-mass findings. The pooled official-weigh-in USG of 1.029 exceeded the commonly applied 1.020 field threshold and approached the range generally interpreted as marked urine concentration. This result is broadly consistent with previous combat-sport hydration syntheses (62). However, USG is influenced by sampling time, recent fluid and solute intake, renal concentration, and methodological variation, and its diagnostic thresholds remain debated in combat-sport settings (19). The seven urine-osmolality and four plasma-osmolality studies could not resolve this uncertainty because their time points and comparisons differed.

Evidence after recovery was insufficient to establish whether hydration was fully restored. Mean USG values of 1.020 before competition and 1.017 at a later post-weigh-in or post-competition assessment each came from a single study. The apparent improvement therefore cannot be interpreted as a pooled recovery effect. Together with the broad osmolality evidence, these findings support a dissociation between weight regain and hydration recovery: athletes may regain several kilograms while still showing concentrated urine or incomplete physiological restoration.

### 4.3 Recovery strategies and exercise performance

Despite the practical importance of post-weigh-in nutrition, no individual recovery strategy had sufficiently homogeneous comparative evidence for a strategy-specific meta-analysis. The evidence map contained seven studies of *ad libitum* intake, five recovery studies of high-carbohydrate meals or refeeding, three studies of carbohydrate-containing beverages, two structured protocols, and single studies of creatine plus glucose, glycerol, caffeine, and sodium citrate; category overlap means that these counts should not be summed. Most intervention categories were represented by small samples and were not independently replicated.

The interventions also differed substantially in composition. Carbohydrate protocols ranged from a 25% glucose-polymer drink providing 1.5 g/kg carbohydrate to 6% beverages providing 60 g/L, including one containing sodium 20 mmol/L and potassium 3.2 mmol/L. The overnight feeding study provided 7.1 g/kg carbohydrate and approximately 4.1 g sodium, whereas the creatine study added 30 g creatine to 320 g glucose. Other studies used glycerol 1 g/kg with 26 mL/kg water, caffeine 6 mg/kg, or sodium citrate 600 mg/kg. This variation makes a generic carbohydrate or electrolyte category clinically imprecise and explains why direct pooling was inappropriate.

Current consensus statements recommend replacing fluid and electrolytes, particularly sodium, and restoring carbohydrate availability after RWL (1, 7). These recommendations are physiologically plausible and consistent with general post-exercise rehydration evidence showing that fluid composition, sodium content, and intake pattern influence fluid retention (63). However, the present review shows that combat-sport-specific comparative evidence remains limited. Consensus recommendations should therefore be understood as best-practice guidance rather than as strategies whose superiority has been established by replicated intervention trials.

Performance evidence was similarly heterogeneous. After multiple outcomes were collapsed within studies, three studies indicated recovery or improvement, six showed mixed findings or no clear difference, and three indicated impairment. This distribution indicates uncertainty rather than equivalence. Small samples, limited statistical power, different tests, and different recovery windows may have obscured meaningful effects. These findings are broadly consistent with Brechney et al. (64), who reported no clear overall difference between pre-RWL and post-recovery performance while identifying reductions in selected outcomes immediately after RWL. Laboratory tests also may not capture competition performance, which depends on tactics, technical skill, opponent characteristics, and psychological state (1).

### 4.4 Practical and regulatory implications

For practitioners, body-mass regain should be treated as one component of recovery monitoring rather than as confirmation of readiness. Where feasible, monitoring should combine body-mass change with information on the magnitude and method of RWL, recovery duration, fluid and sodium intake, gastrointestinal tolerance, hydration markers, symptoms and sport-specific readiness. Recovery plans should be individualized according to whether the preceding mass loss arose predominantly from dehydration, carbohydrate restriction, reduced gastrointestinal contents or a combination of methods.

The results do not support a universal percentage of body mass that every athlete should regain. A higher regain value may reflect a longer recovery window or a larger preceding weight cut rather than more effective recovery. Prescribing a high regain target without considering the original deficit could unintentionally encourage more aggressive RWL. Existing fluid, electrolyte and carbohydrate recommendations remain useful starting points, but should be adjusted to the athlete, competition schedule and possibility of additional weigh-ins.

The findings also have implications for regulation. Longer intervals between weigh-in and competition provide greater recovery opportunities but may encourage larger weight cuts and greater subsequent regain. Moving weigh-ins closer to competition may reduce the incentive for extreme cutting, but could also result in athletes competing while inadequately hydrated if rule changes are not accompanied by minimum-weight certification, education and appropriate hydration monitoring (1, 65). Regulatory approaches should therefore address the magnitude of RWL, rather than simply limiting post-weigh-in recovery.

### 4.5 Strengths and limitations

A major strength of this review was its integration of body-mass, hydration, nutritional-strategy, performance and mechanistic outcomes within a single recovery framework. Assessment periods were kept separate, and relative and absolute body-mass changes were not combined. Random-effects models, prediction intervals, alternative variance estimators, restriction analyses and leave-one-out analyses were used to evaluate robustness. Evidence maps and effect-direction synthesis also allowed heterogeneous strategy and performance data to be retained without forcing clinically incompatible outcomes into pooled estimates.

Several limitations should nevertheless be considered. None of the 40 main studies was judged to be at low risk of bias across all domains. Much of the evidence came from observational or single-arm studies in which RWL, recovery intake and recovery duration were self-selected. Consequently, the pooled body-mass and USG estimates are descriptive and should not be interpreted as causal intervention effects. Heterogeneity was very high, and subgroup analyses were limited by small numbers of studies.

Post-recovery hydration evidence was particularly sparse, while USG measurements varied in timing, protocol and threshold. Performance outcomes were too heterogeneous for quantitative pooling, and the direction-based synthesis did not account fully for effect magnitude or precision. Intervention studies were generally small and rarely replicated independently. Female athletes were underrepresented and concentrated in a small number of cohorts, limiting sex-specific interpretation. Formal assessment of small-study effects was also constrained by the descriptive single-arm design and substantial heterogeneity. Finally, only English-language reports were included, seven potentially relevant reports could not be retrieved, and certainty for non-pooled strategy outcomes was evaluated narratively rather than through outcome-specific GRADE assessments.

### 4.6 Future research

Future studies should distinguish clearly between rapid weight-loss methods and post-weigh-in recovery interventions. Adequately powered randomized or crossover trials should compare standardized fluid, sodium and carbohydrate protocols using consistent doses and recovery windows. Paired analyses should report means, standard deviations and within-participant correlations to permit valid meta-analysis of crossover data.

A core outcome set is also needed. At minimum, studies should report pre-loss body mass, official weigh-in body mass, pre-competition body mass, percentage RWL, percentage regain, recovery duration, fluid and sodium intake, carbohydrate intake, USG or osmolality, adverse events and a reproducible sport-specific performance measure. Studies should also include female athletes, younger athletes and different competition formats. Linking physiological recovery to actual competitive outcomes would help determine whether restoration of body mass, hydration status and substrate availability translates into meaningful improvements in readiness and performance.

## 5 Conclusion

Combat-sport athletes commonly regain substantial body mass after weigh-in, but the pooled means of 6.23% of body mass and 3.71 kg are descriptive averages rather than prescriptive targets because heterogeneity and prediction intervals were large. In five matched cohorts, pooled pre-weigh-in RWL was 8.33% of body mass and the median recovery fraction was 106.5%, although observation windows differed. The pooled official-weigh-in USG of 1.029 indicated concentrated urine at the time of making weight, while post-recovery USG and osmolality data were too sparse or heterogeneous to establish normalization.

Comparative evidence for *ad libitum* intake, carbohydrate-focused recovery, sports drinks, creatine plus glucose, glycerol, caffeine, sodium-containing supplements, and structured protocols was fragmented and did not identify a consistently superior approach. Performance findings were likewise heterogeneous and should be interpreted as uncertain rather than as evidence that recovery strategies have no effect. Recovery should be monitored multidimensionally by considering the preceding weight cut, recovery interval, fluid, sodium and carbohydrate intake, hydration indicators, symptoms, and sport-specific readiness. Adequately powered trials using standardized protocols and a core outcome set are needed, with greater inclusion of female and younger athletes.

## Data Availability

The original contributions presented in the study are included in the article/Supplementary material, further inquiries can be directed to the corresponding author.
