# Supplementary material for: Effects of rehydration and nutritional recovery strategies after rapid weight loss on body mass recovery, hydration status, and performance in combat sports athletes: a systematic review and meta-analysis
Source: Front Nutr. 2026 Sep 18;13:1943678. doi: 10.3389/fnut.2026.1943678 (PMC13630958; doi:10.3389/fnut.2026.1943678)
Supplement: Supplementary file 1 [file Supplementary_file_1.docx]

Supplementary Material

# 1 Supplementary Tables

**Supplementary Table S1.** Search strategies for each database.

| **Database** | **Search strategy** | **Result** |
| --- | --- | --- |
| PubMed | ("Martial Arts"[Mesh] OR "Boxing"[Mesh] OR "Wrestling"[Mesh] OR "combat sport"[tiab] OR "combat sports"[tiab] OR "combat athlete"[tiab] OR "combat athletes"[tiab] OR "martial art"[tiab] OR "martial arts"[tiab] OR "martial artist"[tiab] OR "martial artists"[tiab] OR "mixed martial art"[tiab] OR "mixed martial arts"[tiab] OR MMA[tiab] OR UFC[tiab] OR boxing[tiab] OR boxer*[tiab] OR wrestling[tiab] OR wrestler*[tiab] OR judo[tiab] OR judoka*[tiab] OR taekwondo[tiab] OR "tae kwon do"[tiab] OR "tae-kwon-do"[tiab] OR karate[tiab] OR kickboxing[tiab] OR kickboxer*[tiab] OR "kick boxing"[tiab] OR "muay thai"[tiab] OR "thai boxing"[tiab] OR "Brazilian jiu-jitsu"[tiab] OR "Brazilian jiu jitsu"[tiab] OR "jiu-jitsu"[tiab] OR "jiu jitsu"[tiab] OR jiujitsu[tiab] OR jujitsu[tiab] OR grappling[tiab] OR grappler*[tiab] OR sambo[tiab] OR wushu[tiab] OR "weight class"[tiab] OR "weight classes"[tiab] OR "weight-class"[tiab] OR "weight category"[tiab] OR "weight categories"[tiab] OR "weight-category"[tiab] OR "weight categorized"[tiab] OR "weight-categorized"[tiab] OR "weight-sensitive sport"[tiab] OR "weight-sensitive sports"[tiab]) AND ("Weight Loss"[Mesh] OR "Weight Gain"[Mesh] OR "Body Weight Changes"[Mesh] OR "Dehydration"[Mesh] OR "rapid weight loss"[tiab] OR "rapid weight reduction"[tiab] OR "acute weight loss"[tiab] OR "acute weight reduction"[tiab] OR "weight cutting"[tiab] OR weight-cutting[tiab] OR weight-cut[tiab] OR "making weight"[tiab] OR "make weight"[tiab] OR weigh-in[tiab] OR weighins[tiab] OR "weigh in"[tiab] OR "weigh-ins"[tiab] OR "pre-competition weigh-in"[tiab] OR "post-weigh-in"[tiab] OR "post weigh-in"[tiab] OR "rapid weight gain"[tiab] OR "rapid weight regain"[tiab] OR "weight regain"[tiab] OR "body mass regain"[tiab] OR "body mass gain"[tiab] OR "body mass recovery"[tiab] OR dehydration[tiab] OR dehydrated[tiab] OR hypohydration[tiab] OR hypohydrated[tiab] OR "fluid restriction"[tiab] OR "water loading"[tiab]) AND ("Fluid Therapy"[Mesh] OR "Rehydration Solutions"[Mesh] OR "Water-Electrolyte Balance"[Mesh] OR "Sports Nutritional Sciences"[Mesh] OR rehydration[tiab] OR rehydrate*[tiab] OR rehydrated[tiab] OR "oral rehydration"[tiab] OR "oral rehydration solution"[tiab] OR ORS[tiab] OR electrolyte*[tiab] OR sodium[tiab] OR "fluid replacement"[tiab] OR "fluid intake"[tiab] OR "fluid balance"[tiab] OR "water intake"[tiab] OR drinking[tiab] OR refuel*[tiab] OR refeed*[tiab] OR "re-feed"[tiab] OR "re-fed"[tiab] OR "food intake"[tiab] OR "dietary intake"[tiab] OR carbohydrate*[tiab] OR glycogen[tiab] OR "sports drink"[tiab] OR "sports drinks"[tiab] OR recovery[tiab] OR "recovery time"[tiab] OR "recovery period"[tiab] OR "hydration status"[tiab] OR "urine specific gravity"[tiab] OR USG[tiab] OR "urine osmolality"[tiab] OR "plasma osmolality"[tiab]) NOT (animals[mh] NOT humans[mh]) | 206 |

| **Database** | **Search strategy** | **Result** |
| --- | --- | --- |
| SPORTDiscus (EBSCOhost) | (TX ("combat sport*" OR "combat athlete*" OR "martial art*" OR "martial artist*" OR "mixed martial art*" OR MMA OR UFC OR boxing OR boxer* OR wrestling OR wrestler* OR judo OR judoka* OR taekwondo OR "tae kwon do" OR "tae-kwon-do" OR karate OR kickboxing OR kickboxer* OR "kick boxing" OR "muay thai" OR "thai boxing" OR "Brazilian jiu-jitsu" OR "Brazilian jiu jitsu" OR "jiu-jitsu" OR "jiu jitsu" OR jiujitsu OR jujitsu OR grappling OR grappler* OR sambo OR wushu OR "weight class*" OR "weight-class*" OR "weight categor*" OR "weight-categor*" OR "weight-sensitive sport*")) AND (TX ("rapid weight loss" OR "rapid weight reduction" OR "acute weight loss" OR "acute weight reduction" OR "weight cutting" OR weight-cut* OR "making weight" OR "make weight" OR weigh-in OR weighins OR "weigh in" OR "weigh-ins" OR "pre-competition weigh-in" OR "post-weigh-in" OR "post weigh-in" OR "rapid weight gain" OR "rapid weight regain" OR "weight regain" OR "body mass regain" OR "body mass gain" OR "body mass recovery" OR dehydration OR dehydrated OR hypohydration OR hypohydrated OR "fluid restriction" OR "water loading")) AND (TX (rehydration OR rehydrate* OR rehydrated OR "oral rehydration" OR "oral rehydration solution" OR ORS OR electrolyte* OR sodium OR "fluid replacement" OR "fluid intake" OR "fluid balance" OR "water intake" OR drinking OR refuel* OR refeed* OR re-feed* OR "food intake" OR "dietary intake" OR carbohydrate* OR glycogen OR "sports drink*" OR recovery OR "recovery time" OR "recovery period" OR "hydration status" OR "urine specific gravity" OR USG OR "urine osmolality" OR "plasma osmolality")) | 44 |

| **Database** | **Search strategy** | **Result** |
| --- | --- | --- |
| Web of Science | TS=(( "combat sport*" OR "combat athlete*" OR "martial art*" OR "martial artist*" OR "mixed martial art*" OR MMA OR UFC OR boxing OR boxer* OR wrestling OR wrestler* OR judo OR judoka* OR taekwondo OR "tae kwon do" OR "tae-kwon-do" OR karate OR kickboxing OR kickboxer* OR "kick boxing" OR "muay thai" OR "thai boxing" OR "Brazilian jiu-jitsu" OR "Brazilian jiu jitsu" OR "jiu-jitsu" OR "jiu jitsu" OR jiujitsu OR jujitsu OR grappling OR grappler* OR sambo OR wushu OR "weight class*" OR "weight-class*" OR "weight categor*" OR "weight-categor*" OR "weight-sensitive sport*") AND ( "rapid weight loss" OR "rapid weight reduction" OR "acute weight loss" OR "acute weight reduction" OR "weight cutting" OR weight-cut* OR "making weight" OR "make weight" OR weigh-in OR weighins OR "weigh in" OR "weigh-ins" OR "pre-competition weigh-in" OR "post-weigh-in" OR "post weigh-in" OR "rapid weight gain" OR "rapid weight regain" OR "weight regain" OR "body mass regain" OR "body mass gain" OR "body mass recovery" OR dehydration OR dehydrated OR hypohydration OR hypohydrated OR "fluid restriction" OR "water loading" ) AND ( rehydration OR rehydrate* OR rehydrated OR "oral rehydration" OR "oral rehydration solution" OR ORS OR electrolyte* OR sodium OR "fluid replacement" OR "fluid intake" OR "fluid balance" OR "water intake" OR drinking OR refuel* OR refeed* OR re-feed* OR "food intake" OR "dietary intake" OR carbohydrate* OR glycogen OR "sports drink*" OR recovery OR "recovery time" OR "recovery period" OR "hydration status" OR "urine specific gravity" OR USG OR "urine osmolality" OR "plasma osmolality" )) | 477 |

| **Database** | **Search strategy** | **Result** |
| --- | --- | --- |
| Embase | ('combat sport*' OR 'combat athlete*' OR 'martial art*' OR 'mixed martial art*' OR MMA OR boxing OR boxer* OR wrestling OR wrestler* OR judo OR judoka* OR taekwondo OR karate OR kickboxing OR 'muay thai' OR 'Brazilian jiu-jitsu' OR 'jiu jitsu' OR jiujitsu OR grappling OR sambo OR wushu) AND ('rapid weight loss' OR 'rapid weight reduction' OR 'acute weight loss' OR 'weight cutting' OR 'making weight' OR 'weigh-in' OR weighin* OR 'post-weigh-in' OR 'rapid weight gain' OR 'rapid weight regain' OR 'weight regain' OR 'body mass regain' OR dehydration OR hypohydration OR 'fluid restriction' OR 'water loading') AND (rehydration OR rehydrat* OR 'oral rehydration' OR 'oral rehydration solution' OR ORS OR electrolyte* OR sodium OR 'fluid replacement' OR 'fluid intake' OR refuel* OR refeed* OR carbohydrate* OR glycogen OR 'sports drink*' OR recovery OR 'hydration status' OR 'urine specific gravity' OR USG OR 'urine osmolality' OR 'plasma osmolality') | 253 |

Supplementary Table S2. Characteristics and analytical roles of the 40 main-review studies and 16 additional reports used for supplementary, context-specific, or narrative evidence.

| **Study** | **Evidence role** | **Sex as reported** | **Sport** | **Design** | **Participants** | **Recovery window** | **Exposure or recovery strategy** | **Main outcomes used in this review** |
| --- | --- | --- | --- | --- | --- | --- | --- | --- |
| Scott et al. 1994 [30] | Main recovery synthesis | NR | Collegiate wrestling | Official tournament observation | 668 NCAA wrestlers | ~20 h after weigh-in | Natural post-weigh-in weight regain | Acute weight gain averaged 3.7 +/- 1.3 kg and 4.9 +/- 2.4% BM in non-heavyweights. |
| Oopik et al. 2002 [11] | Main recovery synthesis | Male | Wrestling | Crossover/repeated experimental study | 5 trained wrestlers | 17 h recovery | Glucose vs glucose plus 30 g creatine during recovery | Creatine did not accelerate body-mass recovery, but total work showed a more favourable recovery trend. |
| Finn et al. 2004 [9] | Main recovery synthesis | NR | Wrestling | Intervention experiment | 15 collegiate wrestlers | 1 h after weigh-in | 1.5 g/kg carbohydrate drink vs placebo | Carbohydrate refeeding did not clearly improve repeated sprint power, lactate or mood outcomes. |
| Valiente et al. 2009 [31] | Main recovery synthesis | NR | Wrestling | Randomised crossover rehydration experiment | 21 athletes | 1 h rehydration | Commercially formulated water vs bottled water vs 6% carbohydrate-electrolyte drink | Commercial water was not superior to bottled water or carbohydrate-electrolyte drink for hydration indices. |
| Artioli et al. 2010 [32] | Main recovery synthesis | NR | Judo | Grouped experimental study | 14 judokas; RWL n = 7, control n = 7 | 4 h after weigh-in | Self-selected 5% RWL followed by refeeding/rehydration | Judo-specific performance after 4 h recovery was not worse than control despite lower energy and macronutrient intake. |
| Utter et al. 2010 [10] | Main recovery synthesis | Male | Wrestling | Randomised crossover rehydration experiment | ~21 collegiate wrestlers | 1 h rehydration | Rooibos tea vs bottled water vs 6% carbohydrate beverage | Body-mass regain did not differ between fluids; hydration-marker responses varied by condition. |
| Khamnei et al. 2011 [33] | Main recovery synthesis | Male | Taekwondo | Crossover rehydration experiment | 6 healthy male taekwondo athletes | Immediate voluntary drinking after dehydration | Ad libitum water at 5, 16, 26 or 58 degrees C | Water at 16 degrees C produced the greatest voluntary intake and the smallest fluid deficit. |
| Timpmann et al. 2012 [14] | Main recovery synthesis | NR | Wrestling | Placebo-controlled intervention | 16 trained wrestlers; citrate n = 8, placebo n = 8 | 16 h recovery | Sodium citrate 600 mg/kg vs placebo with ad libitum water | Sodium citrate increased body mass, plasma volume and buffering capacity, but performance and mood changes were similar. |
| Jetton et al. 2013 [16] | Main recovery synthesis | NR | MMA | Real-world repeated-measures study | 40 MMA fighters | ~22 h after weigh-in | Natural acute weight gain and rehydration | Acute weight gain was 3.40 +/- 2.2 kg; USG improved but 39% remained hypohydrated before competition. |
| Mendes et al. 2013 [34] | Main recovery synthesis | Male | Mixed combat sports | Grouped experimental study | 18 male athletes; weight cyclers n = 10, non-weight cyclers n = 8 | 4 h refeeding/rehydration | Up to 5% body-mass loss followed by high-energy/high-carbohydrate recovery | Body mass and lean mass decreased; high-intensity intermittent performance was not clearly impaired after 4 h recovery. |
| Oopik et al. 2013 [35] | Main recovery synthesis | NR | Greco-Roman wrestling | Authentic precompetition repeated-measures study | 51 wrestlers with urine samples; BML n = 27, non-BML n = 24 | Official weigh-in to 16 h recovery | Natural rehydration/refeeding; grouped by body-mass loss status | The BML group regained 2.5 +/- 1.2 kg after 16 h; hypohydration remained common before competition. |
| Lopes-Silva et al. 2014 [13] | Main recovery synthesis | NR | Judo | Randomised crossover intervention | 6 judokas | 4 h recovery after 5-day RWL | Caffeine 6 mg/kg vs placebo during recovery | Caffeine reduced perceived exertion and increased lactate but did not increase throwing performance. |
| Cengiz et al. 2015 [36] | Main recovery synthesis | NR | Wrestling | Real-world precompetition repeated-measures study | 11 elite wrestlers | 12 h meaningful precompetition recovery | Self-selected dehydration and rehydration | Energy/fluid restriction reduced peak power, increased fatigue index and affected heart-rate recovery. |
| Daniele et al. 2016 [37] | Main recovery synthesis | NR | Professional boxing | Retrospective official IBF weigh-in record review | 71 title bouts; 142 boxers | ~12 h between first and second weigh-in | Natural rapid weight gain | Mean regain was 2.52 +/- 1.37 kg and 3.8 +/- 2.2%; weight gain/difference was not clearly associated with bout outcome. |
| Matthews and Nicholas 2017 [38] | Main recovery synthesis | NR | MMA | Real-life fight-preparation case series | 7 MMA athletes | 32 +/- 1 h from weigh-in to fight | Natural RWL/RWG with dietary records | RWL averaged 8.0 +/- 1.8% BM and RWG 11.7 +/- 4.7% BM; dehydration was frequent. |
| Pallares et al. 2016 [39] | Main recovery synthesis | Mixed (124 male, 39 female) | Olympic combat sports | Official competition field repeated-measures study | 163 athletes; 124 men and 39 women | 13-18 h from weigh-in to precompetition | Natural partial rehydration; stratified by urine osmolality | Most athletes were hypohydrated at weigh-in and many remained incompletely recovered before competition. |
| McKenna and Gillum 2017 [12] | Main recovery synthesis | NR | Wrestling | Crossover intervention | 7 male collegiate wrestlers | 60 min rehydration | 26 ml/kg water vs 26 ml/kg water plus 1 g/kg glycerol | After ~3% dehydration, glycerol did not clearly improve acute hydration or anaerobic power. |
| Reale et al. 2017 [40] | Main recovery synthesis | Mixed (70 male, 30 female) | Elite boxing | Real-world multiday tournament observation | 100 elite boxers; 30 women and 70 men | Official weigh-in to 1 h pre-bout | Natural body-mass regain and competition outcomes | Body-mass regain was small and was not associated with winning or tournament progression. |
| Barley et al. 2018 [8] | Main recovery synthesis | NR | MMA | Randomised crossover experiment | 14 MMA athletes | 3 h and 24 h after dehydration | Heat-exercise induced ~5% dehydration followed by ad libitum food and fluid | Repeat-effort performance remained impaired at 3 h and 24 h despite ad libitum recovery. |
| Coswig et al. 2019 [41] | Main recovery synthesis | NR | MMA | Real-world competition observation | 15 MMA athletes; 8 winners and 7 losers | 24 h pre-weigh-in, weigh-in and post-fight | Natural RWL/RWG, dietary intake and technical-tactical analysis | Weight regain, rather than weight loss, was related to selected technical-tactical and outcome variables. |
| Reale et al. 2018 [42] | Main recovery synthesis | NR | Judo/BJJ/wrestling and related grappling sports | Controlled nutrition-strategy experiment | 22 male combat-sport athletes; control n = 10, water loading n = 11 | 3 days water loading/usual intake, 1 day fluid restriction, 2 days recovery | Water loading 100 ml/kg/day vs 40 ml/kg/day before fluid restriction | Water loading produced slightly greater acute weight loss, with no clear performance difference and near-baseline weight after recovery. |
| Zubac et al. 2020 [43] | Main recovery synthesis | Male | Olympic-style boxing | Randomised balanced crossover experiment | 9 elite male boxers | Testing after 3-day 3% RWL; no rehydration window | Low-carbohydrate diet and fluid restriction to induce 3% RWL | RWL reduced MVC, fatigue-task duration and peak lactate and increased USG. |
| Cannataro et al. 2020 [44] | Main recovery synthesis | NR | Muay Thai | Single-arm clinical trial | 21 Italian Muay Thai fighters | RWL followed by RWG | Supervised nutritional RWL/RWG protocol | RWL reduced body mass by 4.1%; most BIA parameters recovered after RWG, with selected endocrine changes. |
| Connor et al. 2020 [45] | Main recovery synthesis | Male | Professional MMA | Crossover/control RWL experiment | 11 male professional MMA athletes | Hot-water immersion after 24 h restriction; next-day recovery | 37.8 degrees C hot-water immersion with fresh water vs 1.6% MgSO4 salt bath | Hot-water immersion reduced body mass; salt bath was not superior to fresh water for RWL or next-morning regain. |
| Brechney et al. 2021 [46] | Main recovery synthesis | NR | MMA | Real-world competition observation/outcome association | 75 MMA athletes; 59 amateur and 16 professional | 7 days pre-fight, official weigh-in and immediate pre-fight | Natural weight cutting and pre-fight body-mass recovery | Greater pre-weigh-in weight loss was associated with lower odds of winning; RWG was similar in winners and losers. |
| Ceylan et al. 2021 [47] | Main recovery synthesis | NR | Judo and wrestling | Competition field repeated-measures study | 27 male judokas and 23 male wrestlers | Official weigh-in to precompetition | Changes in acute weight gain and USG under different weigh-in schedules | Judokas regained more mass than wrestlers, but most athletes remained hypohydrated. |
| Ceylan and Balci 2023 [48] | Main recovery synthesis | NR | Judo | Real-world repeated-measures study | 35 male and 15 female judokas | ~15 h from weigh-in to competition morning | Natural body-mass regain and rehydration | Body mass increased during recovery; USG decreased but most athletes remained markedly hypohydrated. |
| Connor and Egan 2021 [49] | Main recovery synthesis | Male | MMA | Crossover/control RWL experiment | 8 male MMA athletes | Self-adjusted hot-water immersion after 24 h restriction; next-day recovery | Self-selected maximum-tolerable hot-water immersion with fresh water vs 1.6% MgSO4 salt bath | Fresh-water and salt-water immersion produced similar body-mass loss; body mass was largely restored the next day. |
| Kondo et al. 2021 [18] | Main recovery synthesis | Male | Wrestling | Single-arm recovery intervention | 10 elite male wrestlers | 13 h recovery | Three high-carbohydrate meals within 5 h after 6% RWL; total carbohydrate 7.1 g/kg | Body mass and muscle glycogen remained below baseline after overnight recovery. |
| Murugappan et al. 2022 [50] | Main recovery synthesis | NR | Professional boxing | Retrospective official weigh-in record review | 399 male professional boxers | Weigh-in to pre-fight/competition | Natural rapid weight gain after weigh-in | Almost all boxers regained mass; mean regain was 4.4 +/- 2.2 kg and 7.2 +/- 3.5% BM. |
| Ceylan et al. 2022 [17] | Main recovery synthesis | Male | Judo | Controlled experiment | 18 elite male judokas | Post-RWL recovery assessment | 5% RWL within 48 h vs control | RWL increased USG and worsened selected SJFT and heart-rate responses. |
| Ceylan, Barley and Balci 2023 [51] | Main recovery synthesis | Male | Judo | Real-world competition case series | 8 high-level male judokas | Official weigh-in to 24 h later | Natural RWL/RWG and rehydration | Athletes lost 6.8% before weigh-in and regained 4.2% over 24 h; USG improved substantially. |
| Connor et al. 2022 [52] | Main recovery synthesis | Male | MMA | Randomised crossover experiment | 13 male MMA athletes | ~24-26 h high-carbohydrate rehydration/recovery | Fresh hot-water immersion vs 5% magnesium sulphate hot-salt-water immersion after fluid/diet restriction | Total RWL was ~5.3%; hot-water mass loss was similar between baths and performance was not impaired after recovery. |
| Bialowas et al. 2023 [53] | Main recovery synthesis | NR | Judo | Official competition repeated-measures plus questionnaire study | 17 elite judokas | 5 days precompetition to competition | Natural RWL/RWG under IJF rules | RWL prevalence was 100%; urine indices indicated dehydration, but dehydration status was not clearly associated with competition result. |
| Peacock et al. 2023 [54] | Main recovery synthesis | Mixed (50 male, 53 female) | UFC flyweight MMA | Official body-mass record observation | 103 UFC flyweight athletes; 50 men and 53 women | 72/48/24 h pre-weigh-in, official weigh-in and fight weight | Real-world weight loss and fight-day weight regain | Fight weight exceeded official weigh-in weight by 11.4 +/- 3.6% overall, with higher regain in men than women. |
| Donmez et al. 2025 [55] | Main recovery synthesis | NR | Wrestling | Official competition repeated-measures study | 31 Turkish senior wrestlers | Weigh-in to precompetition | Natural dehydration and rehydration | Body composition and USG changed during dehydration and partially recovered during rehydration. |
| Reale et al. 2024 [56] | Main recovery synthesis | NR | Professional MMA | Prospective/retrospective real-world preparation study | 33 athletes across 80 bouts | ~30 h from weigh-in to fight | Dietitian-supported chronic and acute weight-management practices | Early body mass was more predictive of later regain than dehydration in the final 24-48 h. |
| Mauricio et al. 2025 [15] | Main recovery synthesis | Mixed (28 male, 3 female) | MMA | Prospective field intervention/structured preparation | 31 professional MMA athletes; 28 men and 3 women | 30 h after weigh-in | Structured nutrition and fluid manipulation protocol | Athletes lost 10.6% BM over 7 days and regained 11.2% BM within 30 h after weigh-in. |
| Ceylan et al. 2026 [57] | Main recovery synthesis | Male | Kickboxing | Real-world repeated-measures study | 10 elite male kickboxers | Official weigh-in to competition day | Natural RWL/RWG and rehydration | Athletes lost 4.2% BM in the week before weigh-in and regained 4.8%; all remained hypohydrated on competition day. |
| Solarte et al. 2026 [58] | Main recovery synthesis | NR | Boxing/MMA/Muay Thai | Competition field cross-sectional/prediction-model study | 54 amateur combat-sport athletes | ~1-3 h from weigh-in to precompetition | BIA phase/reactance markers and competition outcome | Reactance height-normalised index was associated with outcome models, suggesting possible relevance of rehydration status. |
| Demirkan et al. 2011 [S1] | Supplementary analysis | NR | Cadet wrestling | Training-camp repeated measures | Cadet wrestlers aged 14-17 years | Weigh-in to competition | Natural RWL/RWG | Body mass recovered after weigh-in, whereas USG remained elevated. |
| Yang et al. 2018 [S2] | Supplementary analysis | Male | Taekwondo | Crossover pilot | 5 male athletes | 16 h | 5% rapid versus no weight reduction | Hemorheological markers and simulated performance after recovery. |
| Reljic et al. 2013 [S3] | Supplementary analysis | NR | Amateur boxing | Repeated-measures field study | Elite amateur boxers | Post-RWL recovery time point | Natural RWL and recovery | Body-fluid compartments, blood volume, plasma volume, and haemoglobin mass. |
| Camarço et al. 2016 [S4] | Narrative context | Male | MMA | Case study | 2 professional male athletes | Weigh-in to fight day | Different self-selected RWL exposures | Salivary nitrite, cognition, and upper-body power. |
| Kraemer et al. 2001 [S5] | Supplementary analysis | NR | Wrestling | Simulated tournament | 12 NCAA wrestlers | 2-day simulated tournament | Typical approximately 6% RWL plus matches | Power, strength, hormones, lactate, and plasma osmolality. |
| Pettersson and Berg 2014 [S6] | Supplementary analysis | NR | Mixed combat sports | Competition-day cross-sectional study | 63 elite athletes | Competition day | Hydration observation | Competition-day USG and hypohydration prevalence. |
| Yang et al. 2015 [S7] | Supplementary analysis | Male | Taekwondo | Within-participant intervention | 10 male athletes | Simulated precompetition recovery | 5% rapid versus gradual reduction | Red-cell nitric-oxide signalling and rheology. |
| Ribisl and Herbert 1970 [S8] | Supplementary analysis | NR | Wrestling | Repeated-measures experiment | 8 wrestlers | 5 h | 5% RWL followed by voluntary rehydration | Physical working capacity after loss and rehydration. |
| Shi et al. 2026 [S9] | Supplementary analysis | Male | Boxing | Repeated-measures mechanistic study | 13 male boxers plus 10 young-athlete references | 24 h | RWL and recovery | Serum bile-acid profiles and metabolic pathways. |
| Sagayama et al. 2014 [S10] | Supplementary analysis | Male | Boxing/judo | Single-arm loss-regain study | 10 male weight-category athletes | 12 h | 7-day 5% RWL followed by ad libitum regain | Body composition, body water, and energy expenditure. |
| Sagayama et al. 2019 [S11] | Supplementary analysis | Male | Boxing | Single-arm loss-recovery study | 7 young male boxers | 3 h | RWL followed by an acute recovery meal | Substrate oxidation, energy expenditure, and body mass. |
| Smith et al. 2001 [S12] | Supplementary analysis | NR | Amateur boxing | Dietary/fluid restriction experiment | 8 amateur boxers | 2 days between simulated bouts | Restricted energy and fluid intake | Simulated boxing performance. |
| Houston et al. 1981 [S13] | Supplementary analysis | NR | Freestyle wrestling | Small repeated-measures study | 4 Olympic-level wrestlers | 3 h | RWL followed by fluid and carbohydrate intake | Muscle glycogen and strength. |
| Nishimaki et al. 2018 [S14] | Supplementary analysis | Male | Collegiate wrestling | Randomised crossover RWL-duration study | 9 male athletes | 14 h ad libitum recovery | 1-, 3-, and 7-day 5% RWL | Body water, aldosterone, and oxidative stress. |
| Pereira et al. 2026 [59] | Supplementary analysis | Male | MMA/Muay Thai | Repeated-measures safety study | 30 male athletes | Approximately 24 h | Real-world dehydration and regain | Renal function and urinary markers. |
| Yildirim 2015 [S15] | Supplementary analysis | NR | Wrestling | Precompetition cross-sectional study | 56 elite wrestlers | No recovery window | Precompetition dehydration | Plasma osmolality, cortisol, and testosterone. |

NR, not reported. Sex was recorded exactly as reported by the study and was not inferred from sport or competition category. The first 40 rows comprise the main post-weigh-in recovery review. The remaining 15 reports were used for supplementary or context-specific hydration or prevalence, performance effect-direction, or mechanistic and safety syntheses, and one case study was used only for narrative context. These 16 reports are not represented as included studies in Figure 1. Evidence-role labels identify each report's primary role, whereas contributions to individual outcome syntheses may overlap. Pereira et al. 2026 [59], whose primary role was renal and urinary safety, also provided extractable RWL and body-mass-regain data included in the quantitative body-mass syntheses.

Supplementary Table S3. Timing and body-mass changes in the five matched RWL-RWG cohorts.

| **Study** | **RWL observation window** | **Post-weigh-in follow-up** | **RWL (%BM)** | **RWG (%BM)** | **Loss regained (%)** | **Follow-up relation** | **Timing note** |
| --- | --- | --- | --- | --- | --- | --- | --- |
| Ceylan/Barley/Balci 2023 | One week to official weigh-in | 24 h after competition | 6.89 | 4.35 | 63.2 | Postcompetition | Later measure was not an immediate precompetition value. |
| Mauricio/Artioli 2025 | 7 days | 30 h | 10.56 | 11.25 | 106.5 | Precompetition | Structured weight-management protocol. |
| Matthews and Nicholas 2017 | One week | 32 +/- 1 h | 8.00 | 11.70 | 146.3 | Prefight | Real-world MMA cohort. |
| Peacock et al. 2023 | Final 72 h to official weigh-in | Approximately 24 h | 7.40 | 11.40 | 154.1 | Competition weight | UFC flyweight cohort. |
| Pereira et al. 2026 | Baseline approximately 45 days before; cutting began 8-29 days before weigh-in | Approximately 24 h | 8.85 | 8.40 | 94.9 | Competition day | MMA and Muay Thai cohort. |

RWL, rapid weight loss; RWG, rapid weight gain; %BM, percentage of body mass. Loss regained was calculated as RWG divided by RWL multiplied by 100. Timing was retained as reported and was not harmonised across studies.

Supplementary Table S4. Strategy-outcome evidence and quantitative poolability for recovery, nutrition, and exploratory RWL-method categories.

| **Strategy/category** | **Contributing studies** | **Context/design** | **Body mass/RWG** | **Hydration** | **Performance** | **Mechanistic, safety, or competition outcomes** | **Poolability and evidence conclusion** |
| --- | --- | --- | --- | --- | --- | --- | --- |
| Ad libitum eating and drinking | Mendes et al. 2013 [34]; Artioli et al. 2010 [32]; Barley et al. 2018 [8]; Ribisl and Herbert 1970 [S8]; Nishimaki et al. 2018 [S14]; Reale et al. 2017 [40]; Khamnei et al. 2011 [33] | Natural or prescribed recovery; field, repeated-measures, and small intervention studies | Regain observed, but intake was not standardised | Mixed or incomplete recovery | Mixed findings across sport-specific and repeated-effort tests | USG, fluid deficit, lactate/glucose, and competition outcome were reported inconsistently | Not poolable as a strategy effect; low or very-low evidence |
| High-carbohydrate refeeding or recovery meals | Mendes et al. 2013 [34]; Connor et al. 2022 [52]; Kondo et al. 2021 [18]; Houston et al. 1981 [S13]; Sagayama et al. 2019 [S11]; Zubac et al. 2020 [43] (RWL context) | Recovery meals or carbohydrate-focused protocols with non-equivalent doses and windows | Some regain evidence | Sparse | Mixed or no clear benefit | Muscle glycogen, substrate oxidation, blood glucose, lactate, and urinary markers | Not poolable; five recovery studies plus one RWL-context study |
| Carbohydrate-containing or sports beverages | Finn et al. 2004 [9]; Utter et al. 2010 [10]; Valiente et al. 2009 [31] | Small randomised or crossover rehydration studies using different beverage formulations | Sparse | Mixed; no reproducible advantage over comparator fluids | No clear performance benefit | Plasma/urine osmolality, USG, lactate, and mood | Not poolable because formulations, comparators, and outcomes differed |
| Creatine plus glucose | Oopik et al. 2002 [11] | Single crossover/repeated experiment (n = 5) | Single-study evidence | Metabolic and urinary markers only | No robust benefit established | Ammonia, lactate, glucose, urea, and total work | Not poolable; very-low evidence |
| Glycerol plus water | McKenna and Gillum 2017 [12] | Single crossover study (n = 7) | Not a primary outcome | No clear advantage over water | No clear anaerobic-power benefit | USG and Wingate outcomes | Not poolable; very-low evidence |
| Caffeine | Lopes-Silva et al. 2014 [13] | Single randomised crossover study (n = 6) | Not assessed | Not assessed | No improvement in judo-specific performance | Lower perceived exertion and higher lactate | Not poolable; very-low evidence |
| Sodium citrate | Timpmann et al. 2012 [14] | Single placebo-controlled recovery study (n = 16) | Greater body-mass restoration | Possible plasma-volume, USG, and buffering benefit | No clear performance advantage over placebo | Plasma volume, blood buffering, and mood | Not poolable; very-low evidence |
| Water loading | Reale et al. 2018 [42]; Mauricio et al. 2025 [15] | Controlled RWL experiment and multi-component field protocol | RWL-method evidence rather than isolated post-weigh-in recovery | Urinary electrolyte or fluid-manipulation markers | No reproducible group advantage | Competition and multi-component health outcomes | Not poolable as a post-weigh-in strategy; exploratory/contextual evidence |
| Structured weight-management protocol | Cannataro et al. 2020 [44]; Mauricio et al. 2025 [15] | Multi-component single-arm or prospective field protocols | RWL and RWG observed | Sparse | Sparse | Hormonal, BIA, health-marker, and competition outcomes | Not poolable; the effect of any single nutritional component cannot be isolated |
| Salt versus fresh-water hot bath | Connor et al. 2020 [45]; Connor and Egan 2021 [49] | Two small crossover RWL-method studies | No additional acute body-mass loss with salt; pooled MD -0.04 kg (95% CI -0.52 to 0.44) | Residual urine-osmolality evidence | Not a primary post-weigh-in performance comparison | Blood/plasma-volume markers | Poolable only as an exploratory RWL-method analysis, not as nutritional recovery evidence |

CHO, carbohydrate; MD, mean difference; PV, plasma volume; RWL, rapid weight loss; RWG, rapid weight gain; USG, urine specific gravity. Strategy categories may overlap and counts should not be summed. Evidence conclusions describe the available study set and are not formal GRADE ratings.

# 2 Supplementary Figures

**(A)**

**(B)**

**Supplementary Figure S1**. Domain-level risk-of-bias judgements for the 40 studies in the main recovery synthesis. (A) RoB 2 judgements for randomised and crossover intervention studies. (B) ROBINS-I judgements for non-randomised and observational studies. The 16 supplementary or contextual studies were not included in the overall risk-of-bias summary.

**Supplementary Figure S2.** Funnel plots for relative and absolute body-mass regain after weigh-in. Funnel plots for (A) relative body-mass regain expressed as percentage of body mass and (B) absolute body-mass regain expressed in kilograms. The plots were interpreted descriptively because both syntheses involved single-arm pooled means and substantial between-study heterogeneity.

**Supplementary Figure S3.** Sensitivity analyses for pooled body-mass outcomes.

# 3 Supplementary References

S1. Demirkan E, Koz M, Arslan C, Ersoz G, Kutlu M. The monitoring of weight fluctuation and hydration status in cadet wrestlers (ages 14-17) during a training camp period leading up to competition. Int J Wrestling Sci. (2011) 1:12-18. doi:10.1080/21615667.2011.10878925

S2. Yang WH, Heine O, Grau M. Rapid weight reduction does not impair athletic performance of Taekwondo athletes: a pilot study. PLoS One. (2018) 13:e0196568. doi:10.1371/journal.pone.0196568

S3. Reljic D, Hassler E, Jost J, Friedmann-Bette B. Rapid weight loss and the body fluid balance and hemoglobin mass of elite amateur boxers. J Athl Train. (2013) 48:109-117. doi:10.4085/1062-6050-48.1.05

S4. Camarço NF, Sousa Neto IV, Nascimento DC, Almeida JA, Vieira DCL, Rosa TS, et al. Salivary nitrite content, cognition and power in mixed martial arts fighters after rapid weight loss: a case study. J Clin Transl Res. (2016) 2:63. doi:10.18053/jctres.02.201602.004

S5. Kraemer WJ, Fry AC, Rubin MR, Triplett-McBride T, Gordon SE, Perry Koziris L, et al. Physiological and performance responses to tournament wrestling. Med Sci Sports Exerc. (2001) 33:1367-1378. doi:10.1097/00005768-200108000-00019

S6. Pettersson S, Berg CM. Hydration status in elite wrestlers, judokas, boxers, and taekwondo athletes on competition day. Int J Sport Nutr Exerc Metab. (2014) 24:267-275. doi:10.1123/ijsnem.2013-0100

S7. Yang WH, Heine O, Pauly S, Kim P, Bloch W, Mester J, Grau M. Rapid rather than gradual weight reduction impairs hemorheological parameters of Taekwondo athletes through reduction in RBC-NOS activation. PLoS One. (2015) 10:e0123767. doi:10.1371/journal.pone.0123767

S8. Ribisl PM, Herbert WG. Effects of rapid weight reduction and subsequent rehydration upon the physical working capacity of wrestlers. Res Q. (1970) 41:536-541. doi:10.1080/10671188.1970.10615013

S9. Shi K, Mou D, Luo X, Yang Q. Bile acid profiles reflect metabolic adaptability to rapid weight loss in boxers. BMC Sports Sci Med Rehabil. (2026) 18:200. doi:10.1186/s13102-025-01462-2

S10. Sagayama H, Yoshimura E, Yamada Y, Ichikawa M, Ebine N, Higaki Y, et al. Effects of rapid weight loss and regain on body composition and energy expenditure. Appl Physiol Nutr Metab. (2014) 39:21-27. doi:10.1139/apnm-2013-0096

S11. Sagayama H, Yoshimura E, Yamada Y, Tanaka H. The effects of rapid weight loss and 3-h recovery on energy expenditure, carbohydrate, and fat oxidation in boxing athletes. J Sports Med Phys Fitness. (2019) 59:1018-1025. doi:10.23736/S0022-4707.18.08677-2

S12. Smith M, Dyson R, Hale T, Hamilton M, Kelly J, Wellington P. The effects of restricted energy and fluid intake on simulated amateur boxing performance. Int J Sport Nutr Exerc Metab. (2001) 11:238-247. doi:10.1123/ijsnem.11.2.238

S13. Houston ME, Marrin DA, Green HJ, Thomson JA. The effect of rapid weight loss on physiological functions in wrestlers. Phys Sportsmed. (1981) 9:73-78. doi:10.1080/00913847.1981.11711208

S14. Nishimaki M, Tabata H, Konishi M, Pettersson S, Sakamoto S. Effects of different periods of rapid weight loss on dehydration and oxidative stress. Arch Budo. (2018) 14:319-327.

S15. Yildirim I. Associations among dehydration, testosterone and stress hormones in terms of body weight loss before competition. Am J Med Sci. (2015) 350:103-108. doi:10.1097/MAJ.0000000000000521
